# Event and Comment

**Published:** 1918-12

**Authors:** 


### DENTAL REGISTER

Vol. LXXII DECEMBER, 1918 No. 12

### EVENT AND COMMENT

**The War is Over** For all practical purposes we may feel assured that the terrible war of the past four years, which has taxed the utmost energies and resources of the entire world has ceased. At least the active fighting on an organized national and international basis is at an end. There is a feeling of approaching peace and international fraternity that the world has never before realized, except as an ideal. While it may be true that there are difficulties in the way of readjusting national and international relations, it may be confidently predicted that the spirit of fraternity and liberty for all men that has characterized the cause and enlisted the support of our victorious armies, will predetermine a final harmonious adjustment of all our complex problems. In these last days of the year when we are inclined to "take stock" of our doings for the past year it will be rather difficult to determine exactly what professional progress we may have made in view of the extraordinary distracting as well as exacting events we have had to live with. May we not, however, speculate briefly on the general theme of what the war has done for us as a class of world workers? We may ask first very properly what we have *contributed* directly or even indirectly to the successful prosecution and right outcome of the war; and inversely what has the war brought to dentistry; and lastly what new or added responsibilities has the war and its outcome brought to us? These are three big and it may be difficult questions to answer, but we shall presume to mention a few items that we can assume to be more or less evident to everyone who thinks at all about such things.

**Dentistry's Contribution to the War** We can point with pride to the fact that we have sent into the service in a professional capacity, 5,000 dentists, or enough dentists to place a competent dentist at the disposal of every 500 enlisted soldiers, which means that the enlisted soldiers were much better provided with this kind of care than are the people of the average community in our own country. In the city of Los Angeles, California, there is one dentist for every 1200 inhabitants, and they are all needed. So that we may justly feel that the profession can not be accused of being "slackers," but on the contrary we may be proud of this useful and much needed contribution. Then we may be pardoned if we mention the great work done by the Preparedness League in contributing so generously in the way of free dental service to men who wished to enter the army free from dental deficiencies that might result in their rejection by the examining boards.

Then we should not forget that many dentists who could not go themselves made generous contributions to assist other dentists to go by arranging to take care of their practices until such time as they could return and resume where they had left off. In addition the dentists who remained at home have cheerfully assumed the burden of heavier practice, and uncomplainingly have by careful management assisted the outfitting of the oversea contingent with instruments and materials which they themselves needed badly, but which our manufacturers could not supply because of the shortage of mechanics and materials due to the urgent demands of the army. We need not mention, perhaps, that the profession also assumed its proportion of taxes, bond purchases,

Red Cross and other benevolent demands. We can see by even this cursory review that the dental profession may congratulate itself on its attitude and its substantial support to the carrying on of the war to a satisfactory conclusion.

**What has the War Done for Dentistry** First we can acknowledge our obligation for the wonderful opportunity the war gave us to demonstrate to the world the value of dentistry as an equipment for a good soldier. We have had the chance to show how much better fighting man is the soldier with good teeth. Incidentally we have had a chance to convince our medical associates of the fact that a well man is more largely dependent on good teeth than they have generally believed. The war has given our dentists a chance to acquire unusual skill in surgical and prosthetic treatments and restorations that do not offer in civil practice in ordinary peace times. The experience and training some of our dentists have had opportunity to secure in the field hospitals in association with medical practitioners has been wonderfully rare and beneficial to them. It has brought the medical and dental men into intimate and profitable relations with each other and we are sure that dentists who have been privileged to have an extended experience of this kind will be better prepared for civil practice.

Perhaps one of the most pronounced benefits our profession has acquired during the war period is the legal acknowledgment of the claims our profession has made for many years for an equitable recognition of its professional standing by the United States Government. For years we have been humiliated because Congress refused to give our profession a dignified rank in the army and navy. The war emergencies so clearly demonstrated the professional standing of dentistry that we easily acquired what before had seemed like a distant probability. From these most obvious facts we, as a profession, can feel that while the war has been a great world calamity it has brought to our profession considerable advantages which otherwise would not have materialized so promptly.

**Our New Responsibilities** Since our Government has recognized the profession by a public pronouncement that gives us a position of confidence and trust not only in the army and navy functions, but in all civic life as well, it is evident that the new world relations which our Government has assumed necessitate an extension of our professional work to all the world where our Government and people may carry on active business relations. In brief, we have become a national asset, and we shall be called upon to measure up to the standards of our national as well as our international obligations. We have acquired a new and important responsibility. It may be difficult for us to comprehend at present the scope of this new acquirement. If we take a narrow and selfish view of it, we shall fail disastrously to justify the expectations of those who have honored us, as well as the world which has a right to expect the same high character of professional activity that our Government is carrying to all the world. American dentistry has made an enviable reputation for technical skill both at home and abroad, and we have made much progress in the underlying sciences of our profession. We have in all these directions, much yet to do. We have developed our educational system as far as our knowledge and skill permits, but while we may have placed all the departments of professional activities abreast of the world's attainments, the times demand that we follow our flag in its civilizing career around the world if need be, until we shall have done our share in enlightening the world in our branch of the greatest of all human problems—that of health.

Does our opportunity appeal to us as a great patriotic and benevolent call to a higher service than we have ever dreamed of as a profession; and if so, are we equal to the demands of the occasion? If our country owes a debt of gratitude to France for the heroic sacrifice of La Fayette in our struggle for liberty, does not our profession owe an equally unpaid debt to that great French patriot, Joseph Le Maire, who brought professional dentistry to America in 1780 and established the first training school for dentists in this country in a military camp at Newport, Rhode Island? From that beginning we have developed the highest type of practical and scientific dentistry the world knows, and can we do less now than to perfect our knowledge and skill to our limits that we may return to the old world with interest the inheritance we have for all these years exploited for our own benefit only?

**Veterans of the World's War** The medical men who have been in army service have just organized and incorporated an association of war veterans for the purpose of perpetuating fellowship, preparing medical history of the war, and the securing of mutual benefits for all medical men who have served in the world's war. A large number of influential medical men have acted as incorporators, and undoubtedly it will be one of the important medical societies of the future. Would it be desirable to organize a similar association of dental men, or possibly the dentists may become associate members of the medical organization? No provision appears in the minutes of this new organization for a membership other than of medical men.

**Demobilization** It is not at present known just when the dentists who have enlisted or been drafted will be released from army service, but as a much larger number of commissioned dental officers than were needed to supply the troops at the front, it would seem that the dentists not needed will be released very soon. There is little probability that there will be required in Europe all the American forces now there, and in fact many of the troops not needed at the present time for guard duty, are being sent home. It would seem very probable that we may soon expect a return of the regular order of civil dentistry. There will be needed all the dentists now absent on war business to care for the ever increasing requirements of civil dental practice. It is also probable that we shall not have the great burden of caring for war mutilated dental organs that has been anticipated, owing to the excellent preparatory treatment our enlisted men have had, and also to the fact that the needs of the soldiers in active service have been well looked after. The chances are that our soldiers have had better dental care than the civilians who remained at home, and that our returning soldier dentists will find their patients waiting eagerly for their return.

**Dental Metals Situation Modified**

We have received the following communication from Washington: Washington, November 14. An important step toward a return to pre-war conditions in certain industries was today taken by Van H. Manning, Director of the Bureau of Mines of the Department of the Interior, in modifying the restrictions recently placed on the manufacture and sale of fireworks and of chemicals used in the manufacture of explosives, and on the use of platinum, iridium and palladium in the manufacture of jewelry and for dental and other non-military purposes. Under the order as modified, licenses will no longer be required in connection with the handling of fireworks or the precious metals named. Many of the numerous chemical elements entering into the manufacture of explosives are used constantly for other commercial purposes, and under the new order no restrictions will obtain on the handling of these chemicals when not used in the manufacture of explosives. Licenses are still required for the handling of all such ingredients when intended to be used in explosives manufacture, however.

The Explosives Regulation Act was passed by Congress in October, 1917, with the aim of putting a stop to the numerous incendiary explosions and fires occurring at war munitions plants and other industrial establishments. Under its terms, no one might sell, store or use explosives or ingredients entering into their manufacture without first obtaining a license from the Director of the Bureau of Mines.

The restrictions with regard to platinum, iridium and palladium were added some months later as a means of conserving the nation's limited supplies of these metals, which were essential to the manufacture of explosives and for other war needs, but which were being extensively used in the manufacture of jewelry and for other non-essential purposes.

It is hoped this will clear up the necessity for putting dentists on restricted gold supplies.

**The New Military Dental Course**

The following curriculum is proposed by the *Committee on Education and Special Training*, to be used by dental colleges which adopt military training. In view of the fact that war is about to cease it will probably never be put into effect, for the reason that it schedules too much time.

The division of time suggested will give the total number of hours for class and for laboratory exercises in each subject, also the total hours devoted to each subject, by groups of three terms each year, as follows:

First, Second and Third Terms:

| | Class | Lab. | Total |
|---|---|---|---|
| Biology | 36 | 84 | 120 |
| Inorganic Chemistry | 72 | 144 | 216 |
| Anatomy (arm or leg, thorax, abdomen) | 24 | 96 | 120 |
| Histology (general) | 36 | 84 | 120 |
| Dental Anatomy | 12 | 84 | 96 |
| Operative Technic | 24 | 72 | 96 |
| Prosthetic Technic | 24 | 168 | 192 |
| Technical Drawing | .. | 36 | 36 |
| War Issues and English | 108 | ... | 108 |
| | 336 | 768 | 1104 |
| Study | | | 408 |
| Drill | | | 396 |
| | | | 1908 |

Fourth, Fifth and Sixth Terms:

| | Class | Lab. | Total |
|---|---|---|---|
| Organic Chemistry and Metallurgy | 36 | 84 | 120 |
| Pysiological Chemistry | 24 | 48 | 72 |
| Physics | 24 | 84 | 108 |
| Anatomy (head and neck) | 48 | 144 | 192 |
| Physiology (general) | 60 | 72 | 132 |
| Bacteriology | 36 | 72 | 108 |
| Comp. Dental Anatomy | 12 | ... | 12 |
| Histology (dental) and Embryology | 24 | 36 | 60 |
| Operative Technics | 60 | 108 | 168 |
| Prosthetic Technics | 48 | 216 | 264 |
| Clinic | .. | 60 | 60 |
| | 372 | 924 | 1296 |
| Study | | | 396 |
| Drill | | | 216 |
| | | | 1908 |

Seventh, Eighth and Ninth Terms:

| | Class | Lab. | Total |
|---|---|---|---|
| Physiology (nervous system) | 36 | ... | 36 |
| Materia Medica and Pharmacology | 24 | 48 | 72 |
| Pathology | 48 | 48 | 96 |
| Physical Diagnosis | 12 | 12 | 24 |
| Surgery | 12 | ... | 12 |
| Anesthesia | 12 | ... | 12 |
| Radiography | 12 | ... | 12 |
| Dental Pathology | 24 | 36 | 60 |
| Operative Dentistry | 72 | ... | 72 |
| Prosthetic Dentistry | 48 | ... | 48 |
| Prosthetic Technic | .. | 180 | 180 |
| Orthodontia | 12 | 24 | 36 |
| Oral Hygiene | 24 | ... | 24 |
| Clinic | .. | 576 | 576 |
| | 336 | 924 | 1260 |
| Study | | | 432 |
| Drill | | | 216 |
| | | | 1908 |

Tenth, Eleventh and Twelfth Terms:

| | Class | Lab. | Total |
|---|---|---|---|
| Dental Pathology | 72 | ... | 72 |
| Operative Dentistry | 72 | ... | 72 |
| Prosthetic Dentistry | 72 | ... | 72 |
| Oral Surgery | 72 | 72 | 144 |
| Orthodontia | 36 | ... | 36 |
| Dental Ethics and Jurisprudence | 12 | ... | 12 |
| Dental Economics | 12 | ... | 12 |
| Clinic | .. | 768 | 768 |
| | 348 | 840 | 1188 |
| Study | | | 504 |
| Drill | | | 216 |
| | | | 1908 |

This will make a grand total of 7632 hours' work in the four-year course. Divided as follows: 4848 class, laboratory and clinic hours; 1740 study hours, and 1044 drill hours. It will also mean nine hours each day, or fifty-three hours each week for thirty-six weeks each year. Can any of our dental colleges live up to such a schedule?

